# Kurarinone from *Sophora Flavescens* Roots Triggers ATF4 Activation and Cytostatic Effects Through PERK Phosphorylation

**DOI:** 10.3390/molecules24173110

**Published:** 2019-08-27

**Authors:** Sakiko Nishikawa, Yuka Itoh, Muneshige Tokugawa, Yasumichi Inoue, Ken-ichi Nakashima, Yuka Hori, Chiharu Miyajima, Kou Yoshida, Daisuke Morishita, Nobumichi Ohoka, Makoto Inoue, Hajime Mizukami, Toshiaki Makino, Hidetoshi Hayashi

**Affiliations:** 1 Department of Cell Signaling, Graduate School of Pharmaceutical Sciences and Nagoya City University, Nagoya 467-8603, Japan; 2 Department of Biochemistry, Graduate School of Medicine, University of Yamanashi, Yamanashi 409-3898, Japan; 3 Department of Innovative Therapeutic Sciences, Cooperative Major in Nanopharmaceutical Sciences, Graduate School of Pharmaceutical Sciences, Nagoya City University, Nagoya 467-8603, Japan; 4 Laboratory of Medicinal Resources, School of Pharmacy, Aichi Gakuin University, Nagoya 464-8650, Japan; 5 Division of Molecular Target and Gene Therapy Products, National Institute of Health Sciences, Kanagawa 210-9501, Japan; 6 Department of Pharmacognosy, Graduate School of Pharmaceutical Sciences, Nagoya City University, Nagoya 467-8603, Japan

**Keywords:** ATF4, cancer, ISR, kurarinone, PERK, *Sophora flavescens*, TRB3

## Abstract

In response to cellular stresses, activating transcriptional factor 4 (ATF4) regulates the expression of both stress-relieving genes and apoptosis-inducing genes, eliciting cell fate determination. Since pharmacological activation of ATF4 exerts potent anti-tumor effects, modulators of ATF4 activation may have potential in cancer therapy. We herein attempted to identify small molecules that activate ATF4. A cell-based screening to monitor *TRB3* promoter activation was performed using crude drugs used in traditional Japanese Kampo medicine. We found that an extract from *Sophora flavescens* roots exhibited potent *TRB3* promoter activation. The activity-guided fractionation revealed that kurarinone was identified as the active ingredient. Intriguingly, ATF4 activation in response to kurarinone required PKR-like endoplasmic reticulum kinase (PERK). Moreover, kurarinone induced the cyclin-dependent kinase inhibitor p21 as well as cytostasis in cancer cells. Importantly, the cytostatic effect of kurarinone was reduced by pharmacological inhibition of PERK. These results indicate that kurarinone triggers ATF4 activation through PERK and exerts cytostatic effects on cancer cells. Taken together, our results suggest that modulation of the PERK-ATF4 pathway with kurarinone has potential as a cancer treatment.

## 1. Introduction

The integrated stress response (ISR) is a fine-tuning signaling pathway present in eukaryotic cells that is activated by cells to adapt to a multitude of stresses, including endoplasmic reticulum (ER) stress, glucose deprivation, amino acid deprivation, and hypoxia [1]. In the process of carcinogenesis, the ISR can be triggered by the activation of oncogenes or loss of function in tumor suppressor genes [2,3]. The ISR is initiated by the activation of eukaryotic initiation factor 2α (eIF2α) kinases. There are four members in mammals: double-stranded RNA-dependent protein kinase (PKR), PKR-like ER kinase (PERK), eIF2α kinases heme-regulated inhibitor (HRI), and general control nonderepressible 2 (GCN2) [4]. Once activated, these eIF2α kinases phosphorylate eIF2α, which results in a global decrease in translation. Paradoxically, while the translation of most mRNAs is repressed, several mRNAs are able to bypass this translation block, such as activating transcription factor 4 (ATF4) mRNA, which regulates the transcription of various genes involved in the stress response [5].

ATF4 acts as a master regulator of cellular responses to stress [6]. ATF4 regulates the expression of a broad range of adaptive genes that help cells to withstand periods of stress; however, under persistent stress conditions, cell death pathways are activated. ATF4 induces the expression of proapoptotic effectors, such as C/EBP homologous protein (CHOP), p53 up-regulated modulator of apoptosis (PUMA/BBC3), and phorbol-12-myristate-13-acetate-induced protein 1 (PMAIP1/NOXA). ATF4 is often overexpressed in cancer cells and promotes cell proliferation, survival, and drug resistance [6,7,8]. In contrast, ATF4 activation has been shown to induce tumor cell death under stress conditions [9,10,11]. Since pharmacological activators of the UPR, such as bortezomib, exert potent anti-tumor effects, modulators of ATF4 activation have potential in the treatment of cancer.

Many medicines currently used have many compounds that were originally found in natural organisms. Furthermore, those compounds were developed into more effective and safe synthetic compounds by the addition of various modifications to their molecular structures based on natural chemical structures and the useful pharmacological effects of chemical constituents. Therefore, research to find natural products with new chemical structures and effective biological activities is very important for drug discovery research. We herein attempted to identify low molecular weight compouds that activate ATF4. A cell-based screening to monitor *TRB3* promoter activation, which is a downstream of ATF4 activation, was performed using crude drugs used in traditional Japanese Kampo medicine. Among many drugs, an extract from *Sophora flavescens* roots exhibited potent *TRB3* promoter activation, and kurarinone was identified as their active ingredient. Mechanistically, ATF4 activation in response to kurarinone required PERK. In addition, kurarinone induced the cyclin-dependent kinase (CDK) inhibitor p21 as well as cytostasis in cancer cells. Intriguingly, the cytostatic effect of kurarinone was reduced by pharmacological inhibition of PERK. These results suggest that modulation of the PERK-ATF4 pathway with kurarinone has potential in the treatment of cancer.

## 2. Results

### 2.1. Extract of S. flavescens Roots Induced ATF4 Activation

**Figure 1:**
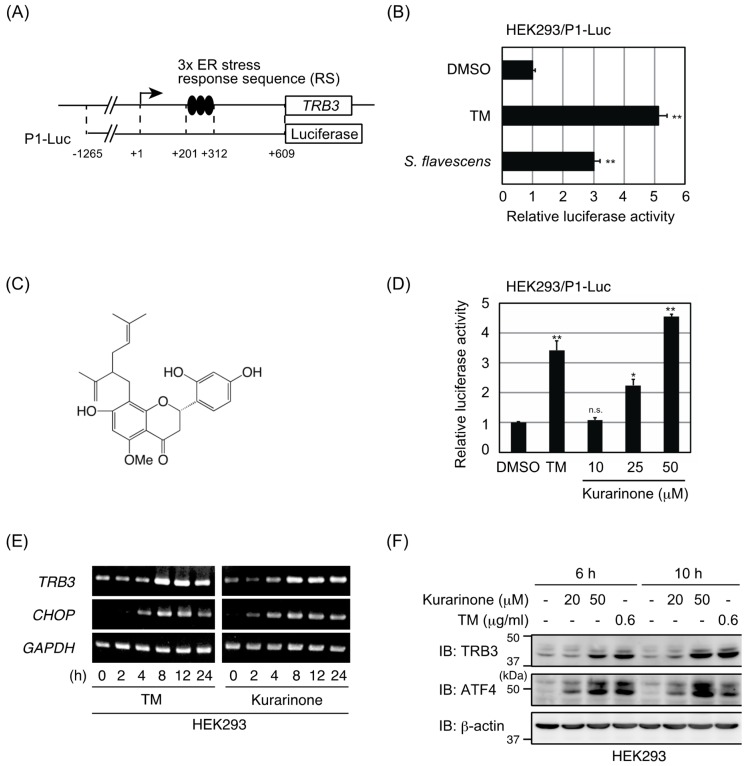
Extract of *Sophora flavescens* roots induced activating transcriptional factor 4 (ATF4) activation. (**A**) A schematic diagram of the human *TRB3* promoter plasmid. (**B**) HEK293/P1-Luc reporter cells were incubated with 2 μg/mL of tunicamycin (TM) or 100 μg/mL of the extract (ex.) of *S. flavescens* roots. After 24 h, luciferase activities were measured. Data represent the mean fold activation ± S.D. (*n* = 3). (**C**) Structure of kurarinone. (**D**) HEK293/P1-Luc reporter cells were incubated with 0.6 μg/mL of TM or the indicated doses of kurarinone. After 24 h, luciferase activities were measured as in (**A**). Data represent the mean fold activation ± S.D. (*n* = 3). (**E**) HEK293 cells were treated with 0.6 μg/mL of TM or 50 μM of kurarinone for the indicated times. The expression level of each gene was assessed by semiquantitative PCR. (**F**) HEK293 cells were incubated with the indicated doses of TM or kurarinone for the indicated periods. The level of the indicated proteins was determined by immunoblotting. Significant differences are indicated as ** *p* < 0.01. * *p* < 0.05. n.s.: not significant.

We previously reported that ATF4 activated the transcriptional activation of *TRB3* in response to a variety of stresses, including ER stress [12]. The *TRB3* promoter contains three tandem 33 base pair repeats and each contains a composite ATF4/CHOP site (ER stress response sequence, Figure 1A) [13]. To identify small molecules that modulate ATF4 activation, we established a HEK293 cell line that stably expresses a human *TRB3* promoter (P1-Luc, Figure 1A). This cell line was confirmed by demonstrating that luciferase activity was induced by the known ER stressor TM (Figure 1B). Subsequently, we screened a library consisting of 119 crude drug extracts that are used in Kampo medicine. We found that the extracts of *S. flavescens* roots and *Notopterygium incisum* roots showed a strong increase in *TRB3* promoter activity (Figure 1B and data not shown). Unfortunately, it has already been shown that falcarindiol contained in the roots of *N. incisum* activates ER stress response [14]. Therefore, we chose *S. flavescens* roots for further investigation.

Although the extract for screening was extracted with methanol (MeOH) alone to evaluate a variety of crude drugs, we changed the extraction solvent to efficiently purify the active ingredient. The dried roots were extracted with acetone to prepare the acetone extract, and then the residue was extracted with MeOH to prepare the MeOH extract. A comparison of these two extracts revealed that *TRB3* promoter activity was markedly induced after exposure to the acetone extract but not the MeOH extract (data not shown). Furthermore, the weight of the acetone extract was much less than that of the methanol extract, suggesting that extraction with acetone would concentrate the active ingredient more. Therefore, the acetone extract was used as the starting material for activity-guided fractionation. The results of activity-guided fractionation of the acetone extract and the isolation of constituents are shown in Figure S1A. Fraction 3, which had the ability to induce ATF4 activation (Figure S1B), was further purified by preparative TLC to obtain the active compound. The compound was identified as kurarinone (Figure 1C) based on EIMS (*m*/*z* 438.52, calcd for C_26_H_30_O_6_^+^, 438.513) and ^1^H and ^13^C-NMR spectroscopic analyses (Figure S2) [15].

### 2.2. Kurarinone Induces TRB3 Expression in an ATF4-Dependent Manner

**Figure 2:**
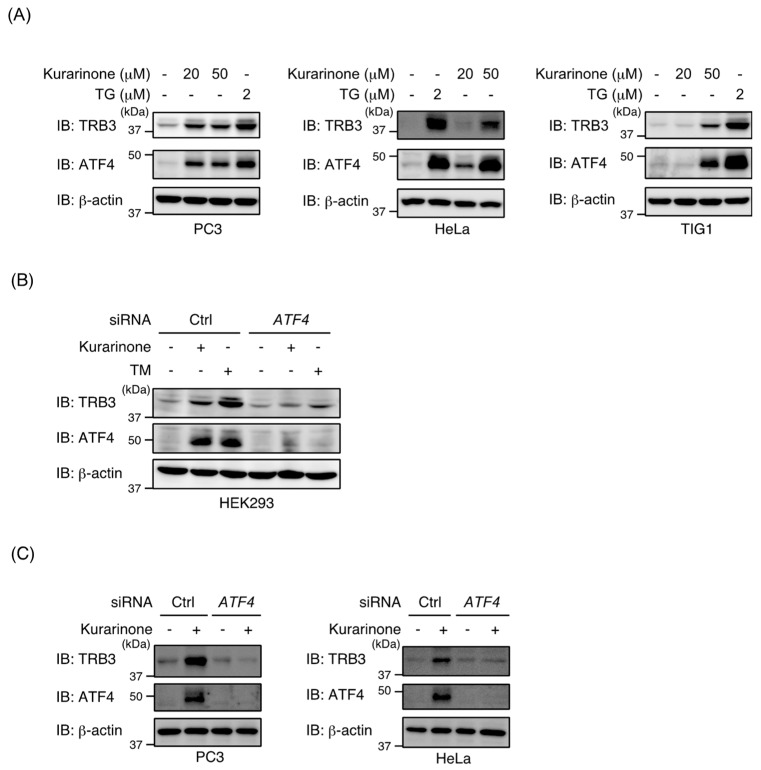
Kurarinone induces *TRB3* expression in an ATF4-dependent manner. (**A**) PC3 cells, HeLa cells, and TIG1 cells were treated with the indicated doses of thapsigargin (TG) or kurarinone for 6 h. Cell lysates were immunoblotted with the indicated antibodies. (**B**) HEK293 cells were transiently transfected with the indicated siRNAs. After 48 h, cells were treated with 0.6 μg/mL of TM or 50 μM of kurarinone for 6 h. Cell lysates were immunoblotted with the indicated antibodies as in (**A**). (**C**) PC3 and HeLa cells were transiently transfected with the indicated siRNAs. After 48 h, cells were treated with 50 μM of kurarinone for 6 h. Cell lysates were immunoblotted with the indicated antibodies as in (**A**). Ctrl, control.

To demonstrate the effects of kurarinone on *TRB3* promoter activity, we performed a reporter assay on HEK293/P1-Luc reporter cells. As shown in Figure 1D, the kurarinone treatment upregulated the promoter activity of *TRB3* in a dose-dependent manner. Kurarinone also up-regulated the expression of *TRB3* and *CHOP* mRNAs as well as that of the TRB3 and ATF4 proteins in HEK293 cells (Figure 1E,F). The induction of TRB3 and ATF4 expression was also observed in PC3 cells (human prostate cancer), HeLa cells (human cervical cancer), and TIG1 cells (normal human diploid fibroblasts) (Figure 2A). To investigate whether ATF4 is required for the upregulation of TRB3 after exposure to kurarinone, ATF4 expression in HEK293 cells was suppressed by siRNA. As shown in Figure 2B, the knockdown of ATF4 inhibited the induction of the TRB3 protein upon the kurarinone treatment. Similar results were obtained using PC3 and HeLa cells (Figure 2C). These results indicate that ATF4 is responsible for the up-regulation of TRB3 by kurarinone.

### 2.3. Kurarinone Triggers ATF4 Activation through the PERK-Eif2α Pathway

**Figure 3:**
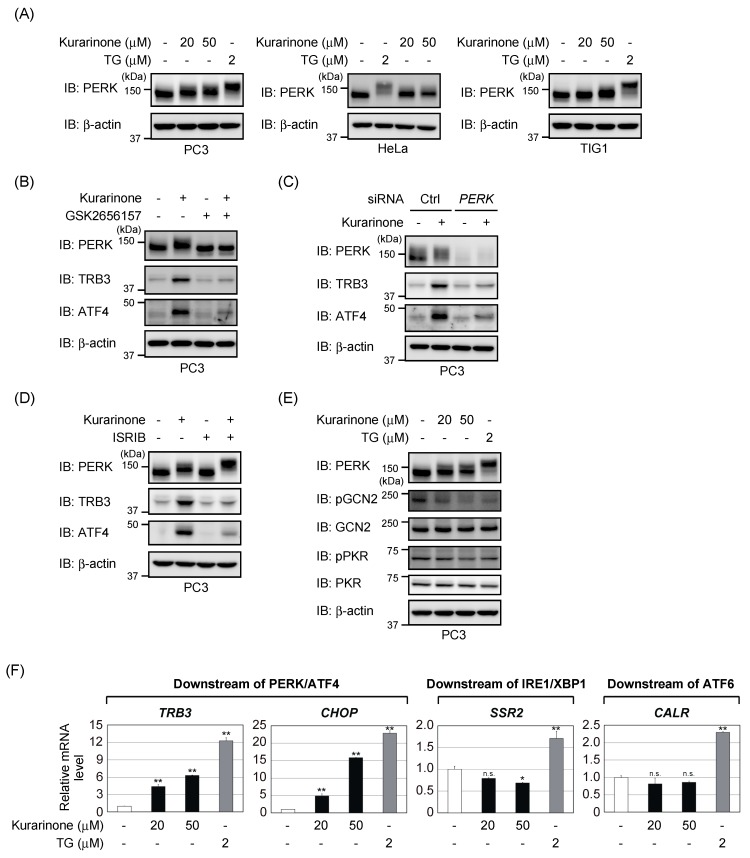
Kurarinone triggers ATF4 activation through the PERK-eIF2α pathway. (**A**) PC3 cells, HeLa cells, and TIG1 cells were incubated with the indicated doses of thapsigargin (TG) or kurarinone for 6 h. The level of the indicated proteins was determined by immunoblotting. (**B**) PC3 cells were pretreated with 1 μM of GSK2656157 for 1 h and then incubated with 50 μM of kurarinone for 6 h. The level of the indicated proteins was determined by immunoblotting as in (**A**). (**C**) PC3 cells were transiently transfected with the indicated siRNAs. After 48 h of transfection, cells were incubated with 50 μM of kurarinone for 6 h. The level of the indicated proteins was determined by immunoblotting as in (**A**). Ctrl, control. (**D**) PC3 cells were pretreated with 0.5 μM of ISRIB for 1 h and then incubated with 50 μM of kurarinone for 6 h. Cell lysates were immunoblotted with the indicated antibodies as in (A). (**E**) PC3 cells were treated with the indicated doses of TG or kurarinone for 6 h. The level of the indicated proteins was determined by immunoblotting as in (A). (**F**) PC3 cells were incubated with the indicated doses of TG or kurarinone for 6 h. The expression level of each gene was assessed by quantitative PCR. Results represent the mean ± S.D. (*n* = 3). Significant differences are indicated as ** *p* < 0.01. * *p* < 0.05. n.s.: not significant.

Since ATF4 is the downstream effector of the ISR, we examined the effects of kurarinone on the initiation of the ISR. A band corresponding to PERK shifted when various cells were incubated with kurarinone (Figure 3A). Since the band was not shifted in the presence of GSK2656157, a catalytic inhibitor of PERK [16], kurarinone appears to have induced PERK phosphorylation (Figure 3B). The induction of ATF4 and TRB3 by kurarinone was also inhibited by GSK2656157 in PC3 cells. The knockdown of PERK also suppressed the induction of ATF4 and TRB3 upon the kurarinone treatment (Figure 3C). These results indicate that kurarinone activates ATF4 through the activation of PERK.

As described above, phosphorylation of eIF2α leads to global translational repression while simultaneously stimulating several mRNAs, including ATF4. A small-molecule ISR inhibitor (ISRIB) was characterized that rescues translation in the presence of phosphorylated-eIF2α by promoting the assembly of more active eIF2B [17,18]. As shown in Figure 3D, the pretreatment of ISRIB suppressed the induction of TRB3 and ATF4 after exposure to kurarinone, indicating that kurarinone activates ATF4 through the PERK-eIF2α pathway. Of note, ISRIB did not suppress, but rather enhanced, the phosphorylation of PERK induced by kurarinone. Although GCN2 or PKR phosphorylate eIF2α in response to various stresses, the treatment with kurarinone failed to induce the phosphorylation of these kinases (Figure 3E).

PERK represents one arm of the UPR, working in conjunction with two other ER-resident membrane proteins, activating transcription factor 6 (ATF6) and inositol-requiring enzyme 1 (IRE1) [1,19,20]. However, we found that kurarinone activated PERK without apparent activation of ATF6 or IRE1. While the treatment with TG induced discriminative target genes expression downstream of the three proteins, kurarinone only induced *TRB3* and *CHOP* mRNAs (downstream of PERK/ATF4) (Figure 3F). Collectively, these results indicate that kurarinone triggers ATF4 activation through the PERK-eIF2α pathway.

### 2.4. Kurarinone Exerts Cytostatic Effects on Cancer Cells

**Figure 4:**
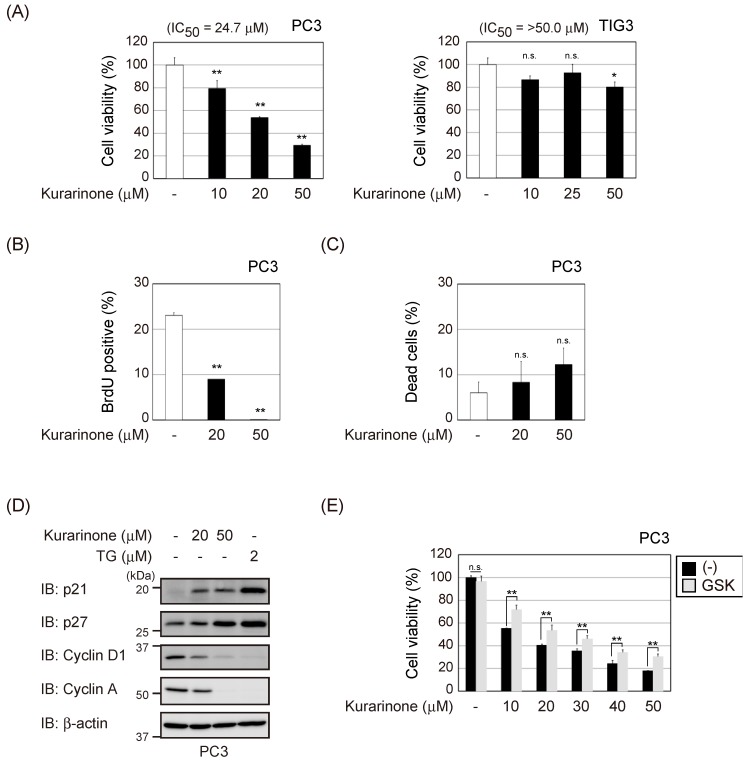
Kurarinone exerts cytostatic effects on cancer cells. (**A**) PC3 and TIG3 cells were incubated with the indicated doses of kurarinone for 48 h. Cell viability was determined by WST-8 assay. Results represent the mean ± S.D. (*n* = 3). (**B**) PC3 cells were incubated with the indicated doses of kurarinone for 48 h. Cells were labeled with 10 μM of BrdU for 1 h and analyzed by fluorescence-activated cell sorting. The average percentage of BrdU-positive cells is shown. Results represent the mean ± S.D. (*n* = 3). (**C**) PC3 cells were exposed to the indicated doses of kurarinone for 48 h. The percentage of dead cells was measured by trypan blue staining. Results were shown as the mean ± S.D. (*n* = 3). (**D**) PC3 cells were incubated with the indicated doses of TG or kurarinone for 6 h. Cell lysates were immunoblotted with the indicated antibodies. (**E**) PC3 and TIG3 cells were exposed to the indicated doses of kurarinone with or without 1 μM of GSK2656157 (GSK) for 48 h. Cell viability was determined by WST-8 assay as in (**A**). Results represent the mean ± S.D. (*n* = 3). Significant differences are indicated as ** *p* < 0.01. * *p* < 0.05. n.s.: not significant.

Kurarinone has been reported to exhibit antitumor activity toward several cancer cells [21,22]. In the present study, it suppressed the proliferation of PC3 cells in a dose-dependent manner (Figure 4A). On the other hand, kurarinone exerted weak toxic effects on normal human diploid fibroblast TIG3 cells (Figure 4A). The selectivity index (SI) [23] of kurarinone between TIG3 cells and PC3 cells was more than 2.02. Thus, it was considered that antiproliferative activity of kurarinone is highly selective for cancer cells. The treatment with kurarinone resulted in less cell death but inhibited G_1_ to S phase progression in PC3 cells (Figure 4B,C). We previously reported that the CDK inhibitor *p21* is a target gene of ATF4 and causes cell cycle arrest after ER stress [24]. Since the induction of p21 causes G_1_ arrest, we investigated whether the treatment with kurarinone upregulated p21 protein expression in PC3 cells. As shown in Figure 4D, kurarinone significantly induced p21 protein expression. Furthermore, the kurarinone treatment reduced the expression of cyclin D1 and cyclin A. These results suggest that p21 upregulated by the PERK-ATF4 pathway contributes to the suppression of PC3 cell proliferation. We also examined whether the cytostatic effects of kurarinone were restored by inhibiting the activity of PERK. The treatment with GSK2656157 significantly recovered the growth arrest induced by kurarinone in PC3 cells (Figure 4E). Collectively, these results indicate that kurarinone triggers ATF4 activation through PERK-eIF2α signaling and exerts cytostatic effects on cancer cells.

## 3. Discussion

In this paper, we demonstrated that kurarinone obtained from the roots of *S. flavescens* activated ATF4. Kurarinone activated PERK located upstream of eIF2α and upregulated the expression of *TRB3* and *CHOP*, which are target genes of the PERK-ATF4 pathway. Moreover, kurarinone induced the CDK inhibitor p21 expression as well as cytostasis in cancer cells. The PERK-ATF4 pathway is recognized as a pathway related to cell cycle arrest and apoptosis in sensors that sense ER stress. Therefore, the development of drug discovery compounds that target the PERK-ATF4 pathway is underway [25,26,27]. Hence, modulation of the PERK-ATF4 pathway with kurarinone has potential as a therapeutic agent for drug discovery, particularly in the treatment of cancer.

*S. flavescens* roots, also known as “Kushen” in Chinese and “Kujin” in Japanese, have traditionally been mainly used in combination with other crude drugs in prescriptions to treat fever, dysentery, hematochezia, jaundice, oliguria, vulvar swelling, asthma, eczema, inflammatory disorders, ulcers, and diseases associated with skin burns [28]. Kurarinone, which is a lavandulyl flavanone, was previously reported to be abundant in *S. flavescens* roots [15,29]. Kurarinone possess numerous pharmacological activities, such as anti-inflammatory [30], anti-microbial [29], and cytotoxic activities against several cancer cells [21,22]. It has also been shown to inhibit the activity of NF-κB [31] and promote TRAIL-induced apoptosis [32]. Previous studies reported that it activated the large-conductance calcium-activated potassium channel and has potential as a therapeutic drug for the treatment of diseases such as overactive bladder syndrome [33,34]. The present results indicate that kurarinone induces the phosphorylation of PERK; however, it failed to activate the IRE1 or ATF6 pathway. The mechanism of activation of PERK by kurarinone needs to be examined in more detail in future studies. In addition, it is necessary to evaluate the physicochemical and absorption, distribution, metabolism and excretion (ADME) properties of kurarinone. Although there are few reports on such properties of kurarinone, it has been reported that kurarinone showed hepatotoxicity [35]. Therefore, it is considered important to create a compound that exhibits anti-cancer activity while eliminating the hepatotoxicity of kurarinone.

In solid cancer, hypoxia and glucose deprivation occur due to the rapid growth of cancer cells and hypoplasia of blood vessels, and an environment with the constitutive activation of UPR is generated [36]. Most cancer cells are necrotic and ultimately die. However, some cancer cells survive and proliferate, thereby avoiding cell death even under this stress. These cancer cells constitutively active UPR and become more resistant to many anti-cancer agents. Therefore, an approach that controls the UPR of cancer cells in the cancer microenvironment is attracting attention as a new cancer therapy. By activating ATF4, research on compounds aimed at killing cancer cells is now being vigorously conducted. The proteasome inhibitor bortezomib triggers ER stress, activates ATF4, and induces cell death. Bortezomib has been reported to exhibit strong cytotoxic activity, particularly against mantle cell lymphoma, which is B-cell malignant lymphoma [37]. Furthermore, ONC201 is a compound currently undergoing clinical trials, and induces the phosphorylation of eIF2α via PKR and HRI to express ATF4 and promote cell death [38,39]. Drug discovery aimed at promoting cancer cells to apoptosis through the induction of ATF4 is also underway.

In conclusion, kurarinone is expected to be a lead compound for new drugs that activate ATF4. Moreover, it may lead to the development of an effective therapeutics for the above-described intractable cancer. Further research is needed to elucidate the precise mode of action of kurarinone underlying the activation of the PERK-ATF4 pathway.

## 4. Materials and Methods

### 4.1. Cell Lines, Plasmids, and RNA Interference

PC3 cells were cultured in Roswell Park Memorial Institute 1640 medium (Sigma, St. Louis, MO, USA) containing 10% fetal bovine serum (FBS) (Sigma), 100 U/mL of penicillin G, and 100 μg/mL of streptomycin [40]. HEK293, HeLa, TIG1, and TIG3 cells were maintained in Dulbecco’s modified Eagle’s medium (Sigma) supplemented with 4.5 g/L glucose, 10% FBS and penicillin/streptomycin [12,24].

*TRB3* promoter P1-Luc (−1265 to +609) was constructed by ligating the human *TRB3* proximal promoter region [12] with pGL4.14 (Promega, Madison, WI, USA). HEK293 cells were transfected using the calcium phosphate method and selected with 100 μg/mL hygromycin.

Regarding short interfering RNA (siRNA) transfection, siRNAs were transfected using Lipofectamine RNAiMAX reagent (Invitrogen, Carlsbad, CA, USA) according to the manufacturer’s instructions. The siRNA oligo targeting human *ATF4* mRNA has been previously described [12]. Human *PERK* siRNA (sense: 5′-CACAAACUGUAUAACGGUU-3′) was obtained from Sigma. Stealth RNAi™ siRNA Negative Control Med GC Duplex was purchased from Invitrogen.

### 4.2. RNA Extraction, Reverse Transcription, and PCR

The RNA extraction was carried out as previously described [41]. The cDNA was synthesized using the PrimeScript first-strand cDNA Synthesis Kit (TaKaRa Bio Inc., Shiga, Japan) [42]. Quantitative PCR was carried out as previously described [42]. Primers used for qPCR were as follows: human *TRB3*, 5′-TGACAACACTTTTCCATGACCATAG-3′ (forward) and 5′-GGAGGCCGACACTGGTACAA-3′ (reverse) [43]; human *CHOP*, 5′-GGTATGAGGACCTGCAAGAGGT-3′ (forward) and 5′-CTTGTGACCTCTGCTGGTTCTG-3′ (reverse) [44]; human *β-actin*, 5′-TGGCACCCAGCACAATGAA-3′ (forward) and 5′-CTAAGTCATAGTCCGCCTAGAAGCA-3′ (reverse) [42]; human *SSR2*, 5′-TTCACCTCGGCAACAATTACT-3′ (forward) and 5′-GGTGCACTGGTAGAGCCAAT-3′ (reverse); human *CALR*, 5′-TGGCGTGCTGGGCCTGGACCTCTGG-3′ (forward) and 5′-AAATGCACCATTTCCTGAGA-3′ (reverse) [45]. Values were normalized by *β-actin*. Semi-qPCR was carried out as previously described [46]. Primers used for semi-qPCR were as follows: human *TRB3*, 5′-TGCCCTACAGGCACTGAGTA-3′ (forward) and 5′-GTCCGAGTGAAAAAGGCGTA-3′ (reverse) [47]; human *CHOP*, 5′-GCGTCTAGAATGGCAGCTGAGTCATTGCC-3′ (forward) and 5′-GCGTCTAGATCATGCTTGGTGCAGATTC-3′ (reverse) [12]; human *GAPDH*, 5′-TGAAGGTCGGAGTCAACGGATTTGGT-3′ (forward) and 5′-CATGTGGGCCATGAGGTCCACCAC-3′ (reverse) [41].

### 4.3. Immunochemical Methods and Antibodies

Immunoblotting was carried out as previously described [48]. Commercially available antibodies used were as follws: anti-p21 (sc-6246; Santa Cruz Biotechnology, Santa Cruz, CA, USA), anti-p27 (610241; BD Biosciences, Franklin Lakes, NJ, USA), anti-cyclin A (611268; BD Biosciences), anti-cyclin D1 (556470; BD Biosciences), anti-ATF4 (11815; Cell Signaling Technology, Beverly, MA, USA), anti-PERK (5683; Cell Signaling Technology), anti-GCN2 (3302; Cell Signaling Technology), anti-TRB3 (ab75846; Abcam, Cambridge, UK), anti-phospho-GCN2 (T899) (ab75836; Abcam), anti-phospho-PKR (T451) (07-886; Sigma) anti-β-actin (A5441; Sigma), and anti-PKR (MAB1980; R&D Systems, Minneapolis, MN, USA).

### 4.4. Luciferase Assay

The luciferase reporter assay was carried out as previously described [41].

### 4.5. Cell Viability Assay and Cell Death Assay

Cell viability was determined using a cell counting kit-8 according to the manufacturer’s protocol (Dojindo, Kumamoto, Japan). Cells were seeded at a density of 5 × 10^3^ cells per well on a 96-well plate. After 24 h, cells were incubated with kurarinone for 48 h. The WST-8 solution was added and cells were incubated at 37 °C for 3 h in a humidified atmosphere of 5% CO_2_. Absorbance of the medium was measured at the wavelength of 450 nm [24]. Regarding the quantification of cell death, cells were stained with trypan blue followed by counting with a hemocytometer under microscope. Stained cells were regarded as dead cells.

### 4.6. BrdU Incorporation Assay

Cells were treated with 10 μM 5-bromo-2′-deoxyuridine (BrdU) at 37 °C for 1 h. BrdU-incorporated cells (S phase) were analyzed with the FITC BrdU Flow kit (BD Biosciences) according to the manufacturer’s protocol. Cells were analyzed using a FACSVerse™ flow cytometer (BD Biosciences).

### 4.7. Preparation of Crude Drug Extracts

The extraction solvent selected for the present study was methanol (MeOH), and 119 crude drugs, which have been used in Japanese traditional Kampo medicine [49], were purchased from Tsumura (Tokyo, Japan). Cut crude drugs (5 g) were stirred in 50 mL of MeOH at room temperature for 24 h and were then filtered using filter paper. This process was repeated three times, and the extracts obtained were pooled. These pooled filtrates were concentrated in vacuo, the residue was dissolved in H_2_O, and the aqueous solution was lyophilized. The lyophilisate was redissolved in dimethyl sulfoxide (DMSO) and stored at −20 °C until used. Screening was performed with crude drug extracts at 100 μg/mL.

### 4.8. Extraction and Isolation of Kurarinone from S. Flavescens Roots

The dried roots of *S. flavescens* (1 kg; Tsumura) were extracted with 3 L of acetone at room temperature for 24 h and then filtered. This process was repeated three times. The filtrate was evaporated under reduced pressure to give an acetone extract (14.8 g). The residue was extracted with 3 L of MeOH, and the filtrate was evaporated to give a MeOH extract (91.3 g). Since an active ingredient was included in the acetone extract, the acetone extract was subjected to SiO_2_ (AP-300: Toyota Kako Co., Ltd., Aichi, Japan) column purification (CHCl_3_/MeOH 20:1 → 10:1 → 0:1) in the stepwise gradient mode and fractions of 100 mL were collected. According to their thin-layer chromatography (TLC, Silica gel 60 F_254_, silica gel RP-18 F_254S_: Merck KGaA, Darmstadt, Germany) profiles, the resulting fractions were combined into six fractions (frs. 1–6). Fr. 3 was further purified by preparative TLC (CHCl_3_/MeOH, 6:1) to obtain an active compound (98 mg).

The structure of the active compound was identified as kurarinone based on comparisons with ^1^H and ^13^C-NMR spectroscopic data in the literature [15]. NMR spectra were recorded using a JNM-AL-400 spectrometer (JEOL Ltd., Tokyo, Japan) with tetramethylsilane as the internal standard. Kurarinone: ^1^H-NMR (acetone-*d_6_*, 400 MHz) *δ* 7.35 (1H, d, *J* = 8.3 Hz), 6.44 (1H, d, 2.4 Hz), 6.41 (1H, dd, *J* = 8.3, 2.4 Hz), 6.16 (1H, s), 5.58 (1H, dd, *J* = 13.2, 2.9 Hz), 4.94 (1H, br t, *J* = 6.8 Hz), 4.53 (1H, d, *J* = 10.2 Hz), 3.69 (3H, s), 2.82 (1H, dd, *J* = 16.6, 13.2 Hz), 2.65 (1H, m), 2.62 (2H, m), 2.51 (1H, m), 2.01 (2H, m), 1.61 (3H, s), 1.52 (3H, s), 1.43 (3H, s); ^13^C-NMR (acetone-*d_6_*, 100 MHz) *δ* 189.9 (C-4), 163.9 (C-7), 162.7 (C-9), 161.2 (C-5), 159.1 (C-4′), 155.9 (C-6′), 149.2 (C-8a), 131.5 (C-5a), 128.4 (C-2′), 124.5 (C-4a), 118.3 (C-1a), 111.1 (C-9a), 108.5 (C-8), 107.7 (C-3′), 106.0 (C-10), 103.4 (C-5′), 93.4 (C-6), 75.0 (C-2), 55.7 (5-OMe), 47.7 (C-2a), 45.6 (C-3), 31.9 (C-3a), 28.0 (C-1a), 25.8 (C-6a), 19.1 (C-10a), 17.8 (C-7a).

### 4.9. Chemicals

Tunicamycin (TM) and thapsigargin (TG) were obtained from Fujifilm-Wako (Osaka, Japan). GSK2656157 and ISRIB were obtained from Cayman Chemical (Ann Arbor, MI, USA). All other chemicals were obtained from Sigma.

### 4.10. Statistical Analysis

The significance of differences between two groups was evaluated using the two-tailed Student’s *t*-test. In multi-group analyses, significance was assessed using a one-way ANOVA with the *post hoc* Tukey-Kramer HSD test.

## Supplementary Materials

The following are available online, Figure S1: The acetone extract of *S. flavescens* roots activated the promoter activity of *TRB3*. Figure S2: ^1^H-NMR spectrum (400 MHz, acetone-*d*_6_) of kurarinone.
