# Supplementary material for: Kurarinone from Sophora Flavescens Roots Triggers ATF4 Activation and Cytostatic Effects Through PERK Phosphorylation
Source: Molecules. 2019 Aug 27;24(17):3110. doi: 10.3390/molecules24173110 (PMC6749437; doi:10.3390/molecules24173110)
Supplement: Supplementary file 1 [file molecules-24-03110-s001.pdf]

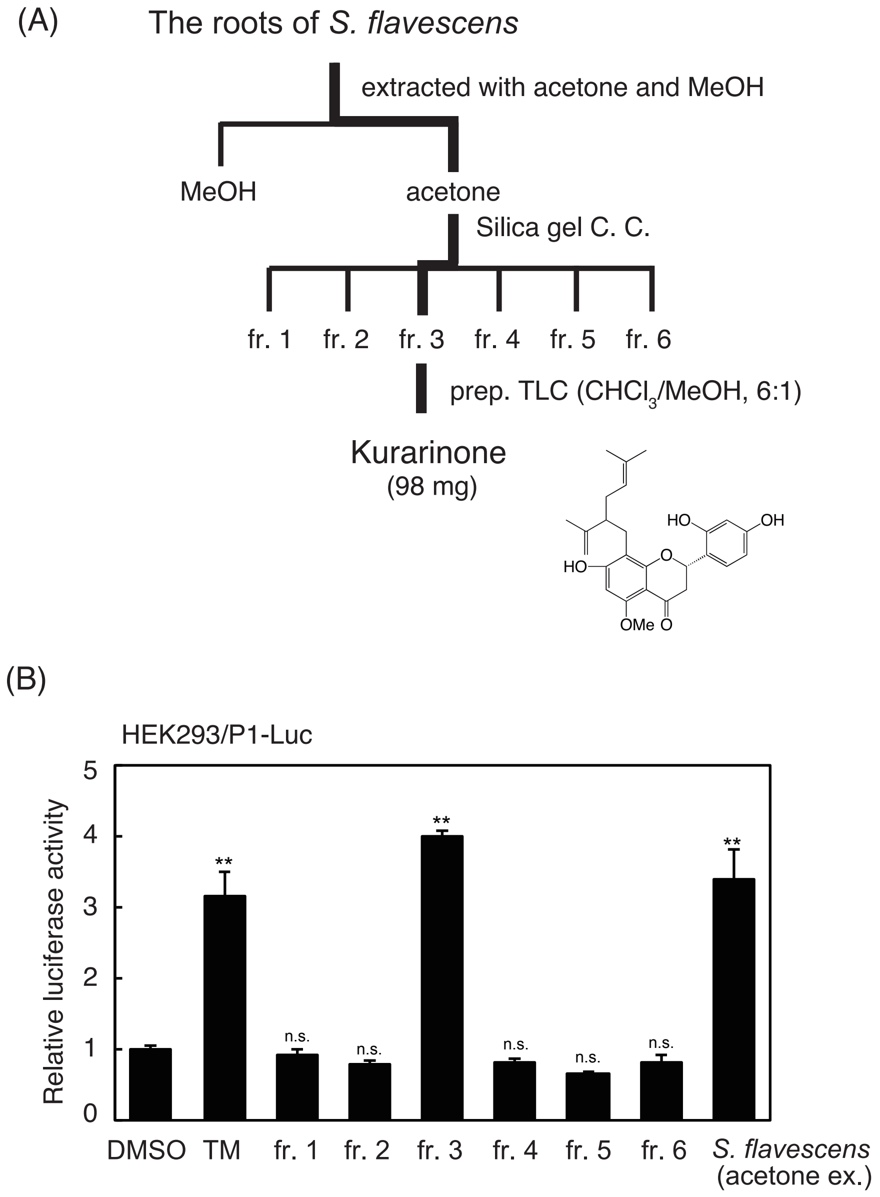

Figure S1. Acetone extract of *S. flavescens* roots activated the promoter activity of TRB3.
(A) Activity-guided fractionation of the acetone extract and isolation of active principles.
(B) HEK293/P1-Luc reporter cells were treated with 0.6 μg/ml of tunicamycin (TM) or 100 μg/ml of the indicated fraction (fr.) of *S. flavescens* roots. After 24 h, luciferase activities in cell lysates were measured. Data are represented as the mean fold activation ± S.D. (n = 3). Significant differences are indicated as $^{**}p < 0.01$. n.s.: not significant.

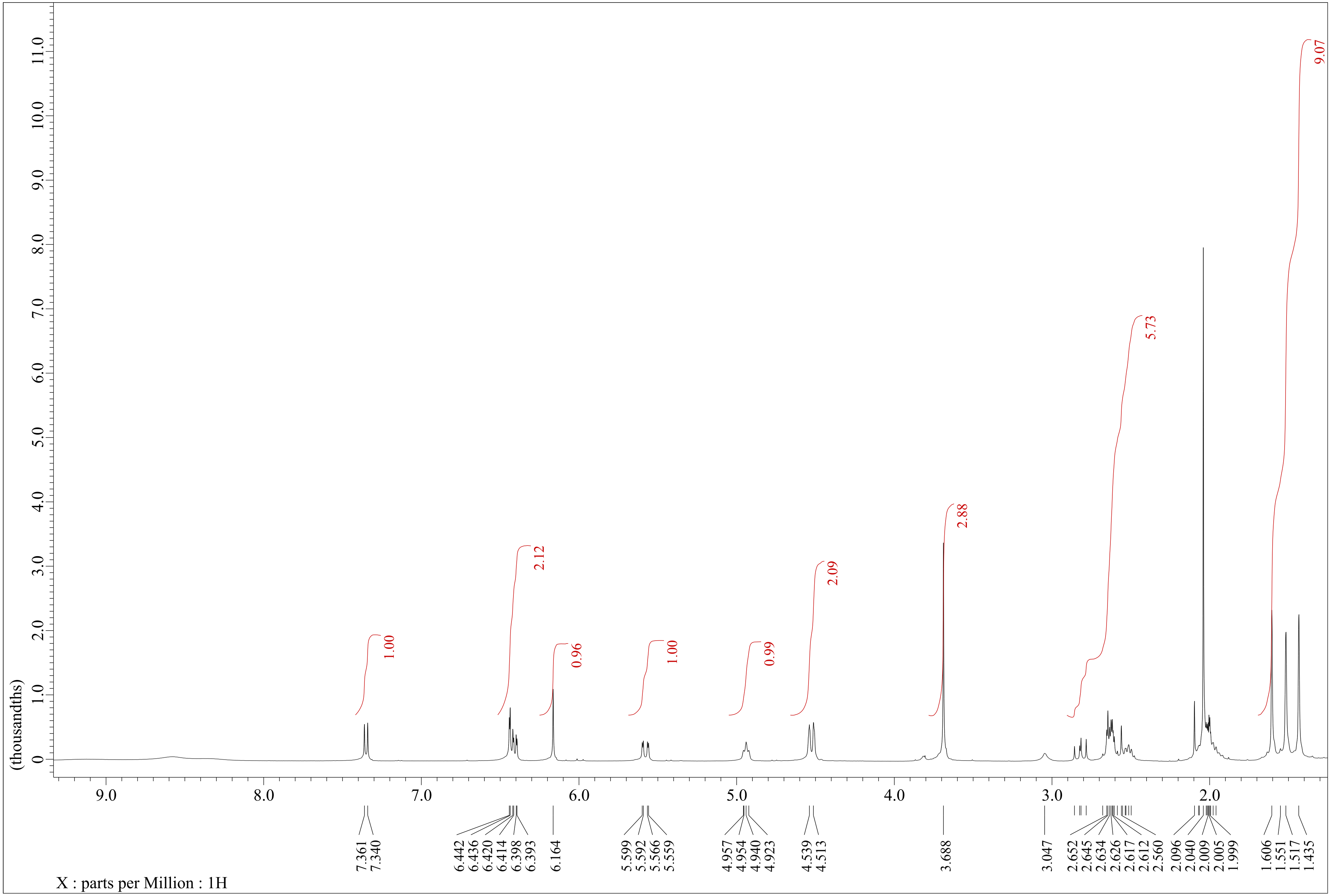

Figure S2. ^{1}H NMR spectrum (400 MHz, acetone-d_6) of kurarinone.
